# Fast Terahertz Imaging Model Based on Group Sparsity and Nonlocal Self-Similarity

**DOI:** 10.3390/mi13010094

**Published:** 2022-01-08

**Authors:** Xiaozhen Ren, Yanwen Bai, Yingying Niu, Yuying Jiang

**Affiliations:** 1 School of Artificial Intelligence and Big Data, Henan University of Technology, Zhengzhou 450001, China; yyjing21@126.com; 2 School of Information Science and Engineering, Henan University of Technology, Zhengzhou 450001, China; baiyw01@126.com

**Keywords:** terahertz imaging, group sparsity, nonlocal self-similarity, conjugate gradient, acceleration scheme

## Abstract

In order to solve the problems of long-term image acquisition time and massive data processing in a terahertz time domain spectroscopy imaging system, a novel fast terahertz imaging model, combined with group sparsity and nonlocal self-similarity (GSNS), is proposed in this paper. In GSNS, the structure similarity and sparsity of image patches in both two-dimensional and three-dimensional space are utilized to obtain high-quality terahertz images. It has the advantages of detail clarity and edge preservation. Furthermore, to overcome the high computational costs of matrix inversion in traditional split Bregman iteration, an acceleration scheme based on conjugate gradient method is proposed to solve the terahertz imaging model more efficiently. Experiments results demonstrate that the proposed approach can lead to better terahertz image reconstruction performance at low sampling rates.

## 1. Introduction

The information obtained from imaging at the Terahertz (THz) frequencies (0.1–10 THz) has received much attention in recent decades due to its unique properties [1,2,3,4,5,6]. Terahertz time domain spectroscopy (THZ-TDS) imaging is one of the most powerful techniques in the terahertz imaging field. It can reconstruct the terahertz image with high signal-to-noise (SNR) ratio and high resolution by scanning the target pixel by pixel. Therefore, it has great application prospects in fields requiring high terahertz imaging quality [7,8,9]. However, THz-TDS imaging technology requires a long time for terahertz image acquisition. Therefore, a fast imaging scheme that can solve the disadvantage of long-time acquisition while maintaining the advantages of THz-TDS detection will attract great interest for practical application.

Numerous techniques have been proposed to solve the problem of long-time acquisition of THz-TDS technology. Terahertz detector arrays have shown great application potential in shortening long-time acquisition [10,11,12], but they suffer from high equipment and complexity costs. A Fourier based terahertz imaging method was proposed to achieve fast terahertz image reconstruction by using sparsity prior to the image [13]. However, it still needs raster scanning on the Fourier plane. Then, a promising terahertz imaging paradigm, namely single pixel imaging system, was proposed [14,15]. This system uses only one detector and avoids raster scanning of the object. Despite the fact that this system could decrease the number of measurements, the speed of switching from one spatial pattern to another is time consuming. Then, various techniques for effective spatial modulation of terahertz beams were studied, but still required additional hardware. Moreover, a block compressive sensing (CS) based method was proposed for terahertz imaging in the spatial domain [16,17], which requires no additional hardware devices and can shorten imaging time. However, a single sparsity prior is utilized in this method for terahertz imaging, and the reconstructed terahertz image quality is not satisfactory. A dual sparsity constraint based spatial domain terahertz imaging strategy was proposed in [18], but may suffer from over-smoothing when the sampling rate is reduced.

In the past few years, a series of nonlocal self-similarity methods have been proposed to extend image denoising from two-dimensional space to three-dimensional space [19,20,21]. These methods use the nonlocal self-similarity of image patches to improve the image quality. A hybrid sparsity model was proposed for terahertz imaging in [22], which utilized the local sparsity and nonlocal self-similarity of the terahertz image to improve image quality, but it does not use the image structure information. Recently, in order to remove noise and reconstruct the image more effectively, methods based on group sparsity have been widely used in image processing, which can extract sparse information from the image structure [23,24,25]. Inspired by the success of group sparsity and nonlocal self-similarity in image processing, a novel fast terahertz imaging model, combined with group sparsity and nonlocal self-similarity (GSNS) is proposed in this paper. It exploits the structure sparsity and similarity of terahertz image patches in both two-dimensional and three-dimensional space to ensure high-quality terahertz image reconstruction, and has the advantages of detail clarity and edge preservation. Furthermore, to overcome the high computational costs of the proposed terahertz imaging model, an acceleration scheme based on a conjugate gradient method is proposed to solve the model more efficiently. Compared with the other methods, the proposed GSNS algorithm can achieve superior performance in fast terahertz imaging.

The rest of the paper is structured as follows. In Section 2, we present the proposed fast terahertz imaging model based on group sparsity and nonlocal self-similarity, and give a solution algorithm using an acceleration scheme in detail. The experiments are utilized to demonstrate the performance of the proposed method in Section 3. Finally, the conclusion is drawn in Section 4.

## 2. Materials and Methods

The THZ-TDS system realizes image reconstruction by scanning each pixel of the target sample. The image acquisition time of THZ-TDS is determined by the number of scanned pixels. Therefore, in order to achieve fast terahertz image reconstruction, we need to reduce the number of scanned pixels. Given a target image $x\in R^{N}$, we randomly select *M* positions from a total of *N* pixels of the target image for terahertz scanning detection. Then the fast terahertz image reconstruction model of the THz-TDS system can be represented as

(1) y=Rx

where $y\in R^{M}$ denotes the measured vector of terahertz data, $x\in R^{N}$ is the target image to be reconstructed, and R denotes the observation matrix composed of 0 and 1 values. The positions of elements with value 1 in the matrix R are determined by the scanning positions of the terahertz system, and the values of other elements are 0.

The purpose of terahertz image reconstruction is to obtain the target image ***x*** from the sparse measured vector ***y***. Since the amount of measured terahertz data is less than the number of pixels in the target image, problem (1) appears ill conditioned. Since the spectral density of an common terahertz image is usually concentrated in the low frequency band and has strong sparsity, imaging methods based on CS can be utilized to reconstruct the terahertz image to reduce the image acquisition time. Based on the sparsity of the terahertz image in frequency domain, the fast terahertz image reconstruction model can be written as

(2) $$\begin{array}{l} \underset{x}{\min}\left\{{\left\|Fx\right\|}_{1}\right\}\;\\ s.t.\;y=Rx \end{array}$$

where *F* is the sparse transformation.

### 2.1. Proposed Fast Terahertz Imaging Model

To further improve the performance of terahertz image reconstruction, a novel fast terahertz imaging model which integrates group sparsity and nonlocal self-similarity constraints is proposed in this section.

Given an image ***x***, we divide it into *P* patches of size *L* × *L*, and the *p*_th_ patch can be denoted by

(3) $$x_{p}=C_{p}x$$

where ***C****_p_* represents the operation of dividing the image into small patches. For each patch ***x****_p_*, we search for its *c*-1 best matching patches in a *T* × *T* search window, and then stack all the best matching patches to form a two-dimensional matrix ***g****_p_* of size *L*^2^ × *c*, where each similar patch is used as a column of ***g****_p_*. Then the matrix ***g****_p_* can be expressed as

(4) $$g_{p}=G_{p}x_{p}=G_{p}C_{p}x$$

where $G_{p}$ is an operator that extracts all patches with similar structures of ***x****_p_* to construct a patch group. For a given dictionary ***D****_p_* learned from the group, the patch group ***g****_p_* can be sparsely expressed as

(5) $$\begin{array}{l} \alpha_{p}=\arg\underset{\alpha_{p}}{\min}{\left\|\alpha_{p}\right\|}_{1}\\ s.t.g_{p}=D_{p}\alpha_{p} \end{array}$$

where $\alpha_{p}$ denotes the sparse coefficient vector. Using the structure similarity and sparsity of the terahertz image, group sparsity could obtain better reconstruction results. After obtaining all the sparse coefficient vectors $\alpha_{p},p=1,2,\ldots P$, the entire image ***x*** can be obtained by [25]

(6) $$x=\sum_{p=1}^{P}G_{p}^{T}\left(D_{p}\alpha_{p}\right)\cdot /\sum_{p=1}^{P}G_{p}^{T}1_{L^{2}\times c}$$

where $1_{L^{2}\times c}$ is a $L^{2}\times c$ matrix and all its elements are 1. $G_{p}^{T}$ is the transpose of ***G****_p_*, which can put a group back to the *p*-th position of the reconstructed image and fill in zeros at other positions. Equation (6) shows that we can recover the image ***x*** by averaging all the overlapped groups.

In addition, motivated by the successful application of nonlocal self-similarity in the three-dimensional transform domain for image denoising, we utilize the nonlocal self-similarity of the terahertz image to explore the structure similarity and sparsity in three-dimensional space in order to ensure high-quality terahertz image reconstruction. Similar to the group sparsity, for a patch ***x****_p_*, we search for its *c*-1 best matching patches in a *T* × *T* search window. However, the difference from group sparsity is that all the best matching patches are stacked to form a three-dimensional matrix ***z****_p_* with the size *L* × *L*×*c*. Then the matrix ***z****_p_* can be expressed as

(7) $$z_{p}=S_{p}x_{p}=S_{p}C_{p}x$$

where $S_{p}$ is an operator that extracts all patches with similar structures of ***x****_p_* to construct a three-dimensional matrix *z_p_*. Let ***T***_3D_ be an orthogonal three-dimensional transformation operator, then the transformation coefficients of the image can be expressed as

(8) $$\Theta_{p}=T_{3D}z_{p}=T_{3D}S_{p}C_{p}x=\Psi_{p}^{3D}x$$

where $\Psi_{p}^{3D}=T_{3D}S_{p}C_{p}$ is the operator of nonlocal self-similarity.

Therefore, nonlocal self-similarity can be used to measure the structure similarity and sparsity of terahertz images in three-dimensional space, which can be expressed as

(9) $$\sum_{p=1}^{P}{\left\|\Theta_{p}\right\|}_{1}=\sum_{p=1}^{P}{\left\|\Psi_{p}^{3D}x\right\|}_{1}$$

In summary, combining group sparsity and nonlocal self-similarity, we propose a new fast terahertz imaging model, which can be expressed as

(10) $$\underset{x}{\min}\sum_{p=1}^{P}\left({\left\|\alpha_{p}\right\|}_{1}+{\left\|\Psi_{p}^{3D}x\right\|}_{1}\right)+\frac{\mu }{2}{\left\|Rx-y\right\|}_{2}^{2}$$

where *μ* is the positive regularization parameter. Obviously, it is extremely difficult to solve the optimization problem (10) directly. Therefore, solving this problem (10) effectively is one of the main contributions of this paper.

### 2.2. Solvution Algorithm Using Acceleration Scheme

Split Bregman iteration can transform a difficult optimization problem into several simple subproblems. Then the subproblems are solved and updated alternately [26,27,28]. Based on the methodology of split Bregman iteration, we introduce $\Theta_{p}=\Psi_{p}^{3D}x$, $\alpha_{p}=D_{p}^{-1}g_{p}=D_{p}^{-1}G_{p}C_{p}x=\Psi_{p}^{2D}x$, Bregman variables $b_{\alpha p}^{}$ and $b_{\Theta p}^{}$, convert the problem (10) into the equivalent split Bregman formula, then the problem (10) can be rewritten as

(11) $$\underset{x,r_{e},\Theta_{p}}{\min}\sum_{p=1}^{P}{\left\|\alpha_{p}\right\|}_{1}+\sum_{p=1}^{P}{\left\|\Theta_{p}\right\|}_{1}+\frac{\mu }{2}{\left\|Rx-y\right\|}_{2}^{2}+\frac{\lambda }{2}\sum_{p=1}^{P}{\left\|\alpha_{p}-\Psi_{p}^{2D}x-b_{\alpha p}\right\|}_{2}^{2}+\frac{\gamma }{2}\sum_{p=1}^{P}{\left\|\Theta_{p}-\Psi_{p}^{3D}x-b_{\Theta p}\right\|}_{2}^{2}\;$$

where *λ* and *γ* are the positive regularization parameters. Then the split Bregman iteration formula of the problem (10) is

(12) $$\begin{array}{l} (x^{i+1},\alpha_{p}^{i+1},\Theta_{p}^{i+1})=\arg\underset{x,\alpha_{p},\Theta_{p}}{\min}\sum_{p=1}^{P}{\left\|\alpha_{p}\right\|}_{1}+\sum_{p=1}^{P}{\left\|\Theta_{p}\right\|}_{1}+\frac{\mu }{2}{\left\|Rx-y\right\|}_{2}^{2}\\ \;\;\;\;\;\;\;\;\;\;+\frac{\lambda }{2}\sum_{p=1}^{P}{\left\|\alpha_{p}-\Psi_{p}^{2D}x-b_{\alpha p}^{i}\right\|}_{2}^{2}+\frac{\gamma }{2}\sum_{p=1}^{P}{\left\|\Theta_{p}-\Psi_{p}^{3D}x-b_{\Theta p}^{i}\right\|}_{2}^{2} \end{array}$$

and the Bregman updates are

(13) $$\left\{\begin{array}{c} b_{\alpha p}^{i+1}=b_{\alpha p}^{i}+(\Psi_{p}^{2D}x^{i+1}-\alpha_{p}^{i+1})\\ b_{\Theta p}^{i+1}=b_{\Theta p}^{i}+(\Psi_{p}^{3D}x^{i+1}-\Theta_{p}^{i+1}) \end{array}\right.$$

The *l*_1_ norm in the optimization problem (12) is not differentiable, so it is still difficult to solve directly. Based on the strategy of separating variables, we convert the optimization problem (12) into three subproblems, and iteratively update each variable by fixing other variables.

***x*** subproblem: fixing $\alpha_{p}$,$\Theta_{p}$,$b_{\alpha p}^{}$ and $b_{\Theta p}^{}$, the subproblem of updating ***x*** can be given from (12)

(14) $$\begin{array}{l} x^{i+1}=\underset{x}{\min}Q_{1}(x)\\ \;\;\;\;\;\;\;=\underset{x}{\min}\frac{\mu }{2}{\left\|Rx-y\right\|}_{2}^{2}+\frac{\lambda }{2}\sum_{p=1}^{P}{\left\|\alpha_{p}^{i}-\Psi_{p}^{2D}x-b_{\alpha p}^{i}\right\|}_{2}^{2}+\frac{\gamma }{2}\sum_{p=1}^{P}{\left\|\Theta_{p}^{i}-\Psi_{p}^{3D}x-b_{\Theta p}^{i}\right\|}_{2}^{2}\; \end{array}$$

The subproblem (14) is a minimization problem of typical convex function. Using the differential for $Q_{1}(x)$ and setting the result equal to zero, we can obtain

(15) $$⟮\mu R^{T}R+\lambda \sum_{p=1}^{P}{\left(\Psi_{p}^{2D}\right)}^{T}\Psi_{p}^{2D}+\gamma \sum_{p=1}^{P}{\left(\Psi_{p}^{3D}\right)}^{T}\Psi_{p}^{3D}⟯x^{i+1}=\mu R^{T}y+\lambda \sum_{p=1}^{P}{\left(\Psi_{p}^{2D}\right)}^{T}\left(\alpha_{p}^{i}-b_{\alpha p}^{i}\right)+\gamma \sum_{p=1}^{P}{\left(\Psi_{p}^{3D}\right)}^{T}\left(\Theta_{p}^{i}-b_{\Theta p}^{i}\right)$$

Then, ***x*** can be updated as

(16) $$x^{i+1}={⟮\mu R^{T}R+\lambda \sum_{p=1}^{P}{\left(\Psi_{p}^{2D}\right)}^{T}\Psi_{p}^{2D}+\gamma \sum_{p=1}^{P}{\left(\Psi_{p}^{3D}\right)}^{T}\Psi_{p}^{3D}⟯}^{-1}z^{i}$$

where

(17) $$z^{i}=\mu R^{T}y+\lambda \sum_{p=1}^{P}{\left(\Psi_{p}^{2D}\right)}^{T}\left(\alpha_{p}^{i}-b_{\alpha p}^{i}\right)+\gamma \sum_{p=1}^{P}{\left(\Psi_{p}^{3D}\right)}^{T}\left(\Theta_{p}^{i}-b_{\Theta p}^{i}\right)$$

$\Theta_{p}$ subproblem: fixing x,$\alpha_{p}$,$b_{\alpha p}^{}$ and $b_{\Theta p}^{}$, the subproblem of updating $\Theta_{p}$ can be given from (12)

(18) $$\Theta_{p}^{i+1}=\underset{\Theta_{p}}{\min}\sum_{p=1}^{P}\left({\left\|\Theta_{p}\right\|}_{1}+\frac{\gamma }{2}{\left\|\Theta_{p}-\Psi_{p}^{3D}x^{i+1}-b_{\Theta p}^{i}\right\|}_{2}^{2}\right)$$

By introducing the soft thresholding algorithm, each $\Theta_{p}$ can be updated as [29]

(19) $$\Theta_{p}^{i+1}=\mathrm{shrink}\left(\Psi_{p}^{3D}x^{i+1}+b_{\Theta p}^{i},1/\gamma \right)$$

where

(20) $$\begin{array}{l} \mathrm{shrink}\left(x,\lambda \right)=\mathrm{sgn}(x)\times \max\left(\left|x\right|-\lambda ,0\right)\\ \;\;\;\;\;\;\;\;\;\;\;\;\;\;\;\;\;\;\;=\left\{\begin{array}{c} x-\lambda ,\\ 0,\\ x+\lambda , \end{array}\right.\;\begin{array}{c} x\in \left(\lambda ,+\infty \right)\\ x\in \left(-\lambda ,\lambda \right)\\ \;x\in \left(-\infty ,-\lambda \right) \end{array} \end{array}$$

$\alpha_{p}$ subproblem: Fixing x,$\Theta_{p}$,$b_{\alpha p}^{}$ and $b_{\Theta p}^{}$, the subproblem of updating $\alpha_{p}$ can be given from (12)

(21) $$\alpha_{p}^{i+1}=\underset{\alpha_{p}}{\min}\sum_{p=1}^{P}⟮{\left\|\alpha_{p}\right\|}_{1}+\frac{\lambda }{2}{\left\|\alpha_{p}-\Psi_{p}^{2D}x-b_{\alpha p}^{i}\right\|}_{2}^{2}⟯$$

Similar to the optimization problem (18), each $\alpha_{p}$ can be updated by the soft thresholding algorithm.

(22) $$\alpha_{p}^{i+1}=\mathrm{shrink}\left(\Psi_{p}^{2D}x^{i+1}+b_{\alpha p}^{i},1/\lambda \right)$$

Then, these simple subproblems are solved iteratively to obtain the reconstructed terahertz image.

Although the optimization problem (12) can be solved by iterating three subproblems (16), (18) and (21), the computational complexity is still very high. The main computational complexity comes from solving the ***x*** subproblem because the cost of computing the matrix inversion is too high, which is *O*(*N*^3^). So, an effective method is highly desirable. Conjugate gradient method is an effective strategy for solving large-scale optimization problems, which has a simple iterative form [30,31]. The basic concept of the conjugate gradient method is to combine the conjugate property with the steepest descent method, construct a set of conjugate directions by using the gradient at known points, search along this set of directions, and find the minimum point of the objective function. Therefore, an acceleration scheme based on the conjugate gradient method is proposed to tackle the ***x*** subproblem.

For convenience, we let

(23) $$A=\mu R^{T}R+\lambda \sum_{p=1}^{P}{\left(\Psi_{p}^{2D}\right)}^{T}\Psi_{p}^{2D}+\gamma \sum_{p=1}^{P}{\left(\Psi_{p}^{3D}\right)}^{T}\Psi_{p}^{3D}$$

(24) $$z=\mu R^{T}y+\lambda \sum_{p=1}^{P}{\left(\Psi_{p}^{2D}\right)}^{T}\left(\alpha_{p}^{i}-b_{\alpha p}^{i}\right)+\gamma \sum_{p=1}^{P}{\left(\Psi_{p}^{3D}\right)}^{T}\left(\Theta_{p}^{i}-b_{\Theta p}^{i}\right)$$

(25) $$u=x^{i+1}$$

Then, (15) can be rewritten as

(26) Au=z

Therefore, the solution of the Equation (26) can be equivalent to

(27) $$\underset{u}{\min}f(u)=\underset{u}{\min}\frac{1}{2}u^{T}\mathrm{Au}-z^{T}u\;$$

It can be seen that the optimization problem (27) is a quadratic programming problem, which can be solved by the conjugate gradient method.

Firstly, given an initial point ***u***^0^, calculate the gradient of the objective function *f* (***u***) at this point ***u***^0^ and set the first search direction ***d***^0^ as the initial negative gradient direction

(28) $$d^{0}=-\nabla f(u^{0})=-g^{0}$$

Search along direction ***d***^0^ to obtain the next iteration point ***u***^1^. Calculate the gradient ***g***^1^ of *f* (***u***) at ***u***^1^. If ||***g***^1^|| ≠ 0, use ***g***^1^ and ***d***^0^ to construct the second search direction ***d***^1^, and search along ***d***^1^ to obtain point ***u***^2^. Generally, if the point ***u****^k^* and the search direction ***d****^k^* are known, we search along ***d****^k^* starting from ***u****^k^* and get the next iteration point

(29) $$u^{k+1}=u^{k}+\alpha^{k}d^{k}\;$$

where $\alpha^{k}$ is step size, satisfying

(30) $$\alpha^{k}=\arg\underset{\alpha^{k}}{\min}f(u^{k}+\alpha^{k}d^{k})$$

Solve (30) to obtain

(31) $$\alpha^{k}=\frac{{\left(g^{k}\right)}^{T}d^{k}}{{\left(d^{k}\right)}^{T}Ad^{k}}\;$$

and the gradient ***g****^k^* ^+ 1^ of the objective function *f* (***u***) at the current iteration point ***u****^k^* ^+ 1^ is

(32) $$g^{k+1}=\nabla f(u^{k+1})=g^{k}-\alpha^{k}Ad^{k}\;$$

Then, a new direction ***d****^k^* ^+ 1^ orthogonal to the previous search direction is constructed by using the linear combination of the previous search direction ***d****^k^* and the gradient ***g****^k^* ^+ 1^ of the objective function *f* (***u***) at the current iteration point ***u****^k^* ^+ 1^

(33) $$d^{k+1}=g^{k+1}+\beta^{k}d^{k}\;$$

According to the conjugation of ***d****^k^* ^+ 1^ and ***d****^k^* with respect to ***A***, we get

(34) $$\beta^{k}=\frac{{\left(g^{k+1}\right)}^{T}g^{k+1}}{{\left(g^{k}\right)}^{T}g^{k}}\;$$

When the iteration ends, we get the solution $x^{i+1}$ of the ***x*** subproblem. The main computational complexity of the conjugate gradient method comes from the multiplication of matrix and vector, which is *O*(*N*^2^). Therefore, the computational complexity can be reduced by using the conjugate gradient method instead of matrix inversion.

To sum up, we introduce the acceleration scheme into the split Bregman iterative framework, and obtain the main steps of the proposed algorithm summarized in Algorithm 1.
**Algorithm 1** The proposed algorithm by combining group sparsity and nonlocal self-similarity.**Input:**   measured terahertz data ***y***, observation matrix ***R***, sparsity basis $\Psi_{p}^{2D}$, nonlocal self-similarity operator $\Psi_{p}^{3D}$.**Initialization:**   $\alpha_{p}^{0}=\Theta_{p}^{0}=b_{\alpha p}^{0}=b_{\Theta p}^{0}=0$, *μ*, *λ*, *γ*, estimate an initial image ***x***^0^.**Loop:** set *i* = 0 and repeat until $||x^{i+1}-x^{i}|{|}_{2}<\delta$  $g^{0}=z^{i}-Ax^{i}$  $d^{0}=-g^{0}$  k=0  while  $\alpha^{k}=\frac{{\left(g^{k}\right)}^{T}d^{k}}{{\left(d^{k}\right)}^{T}Ad^{k}}$  $u^{k+1}=u^{k}+\alpha^{k}d^{k}$  $g^{k+1}=g^{k}-\alpha^{k}Ad^{k}$  if $||g^{k+1}||<\varepsilon$, break   $\beta^{k}=\frac{{\left(g^{k+1}\right)}^{T}g^{k+1}}{{\left(g^{k}\right)}^{T}g^{k}}$  $d^{k+1}=g^{k+1}+\beta^{k}d^{k}$  k=k+1  return $u^{k+1}$  $x^{i+1}=u^{k+1}$  $\Theta_{p}^{i+1}=\mathrm{shrink}\left(\Psi_{p}^{3D}x^{i+1}+b_{\Theta p}^{i},1/\gamma \right)$  $\alpha_{p}^{i+1}=\mathrm{shrink}\left(\Psi_{p}^{2D}x^{i+1}+b_{\alpha p}^{i},1/\lambda \right)$  $b_{\alpha p}^{i+1}=b_{\alpha p}^{i}+(\Psi_{p}^{2D}x^{i+1}-\alpha_{p}^{i+1})$  $b_{\Theta p}^{i+1}=b_{\Theta p}^{i}+(\Psi_{p}^{3D}x^{i+1}-\Theta_{p}^{i+1})$  i=i+1**End Loop****Output:*****x***


## 3. Experiments and Discussion

The THz-TDS reflection imaging system with a measurement range of 5 × 5 cm was used in the experiment. The terahertz pulse is generated by the laser with a pulse width of 100 fs and a repetition rate of 80 MHz. The terahertz beam generated by pump beam is focused onto the sample; then the beam reflected from the sample is sent to a ZnTe crystal, where it overlaps with the probe beam. The probe beam is modulated by the terahertz field and then is guided to the detector [18]. During the imaging process, the sample is moved in a mechanical grating scan, and the spatial resolution is set to 0.25 mm. By programming the scan positions determined by the observation matrix, we could easily obtain the proposed fast terahertz imaging system from a traditional THz-TDS system.

**Figure 1:**
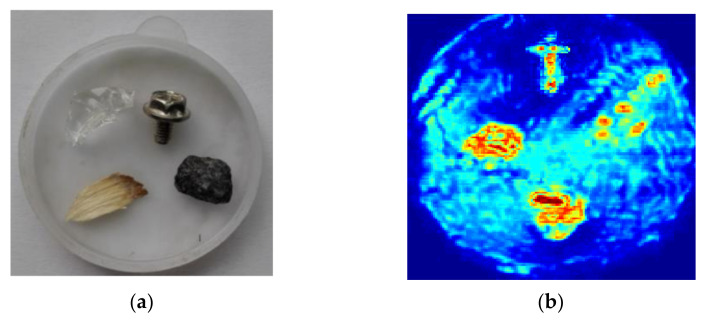
The sample and the terahertz image obtained through full scan imaging process. (**a**) sample; (**b**) terahertz image.

In the experiment, a glass fragment, a metal screw, a wood chip and a stone embedded in flour with a depth of 10 mm were measured by THz-TDS system at room temperature. Figure 1a shows the sample placed in a 3.5 cm diameter dish, and Figure 1b presents the terahertz image of the sample obtained from the reflection of four objects and flour through full scan imaging process. The terahertz image of the sample is obtained at 0.8 THz and the number of the full scan pixels is 150 × 150. In the experiment, all parameters are set empirically. The size of the patches is 8 × 8. The horizontal or vertical distance between two adjacent patches is 4. The number of best matching patches is set to 10 and the search window is set to 40 × 40. To quantitatively evaluate the imaging performance of terahertz images, peak signal-to-noise ratio (PSNR) [32] and relative *l*_2_ norm error (RLNE) [33] are used as image quality assessment indexes in this paper, which are

(35) $$\mathrm{PSNR}=10\log_{10}\frac{peakval^{2}}{\mathrm{MSE}(x,\hat{x})}$$

(36) $$\mathrm{RLNE}=\frac{{\left\|x-\hat{x}\right\|}_{2}}{{\left\|x\right\|}_{2}}$$

where peakval denotes the peak value of the terahertz image, and $\mathrm{MSE}(x,\hat{x})$ denotes the mean square error.

**Figure 2:**
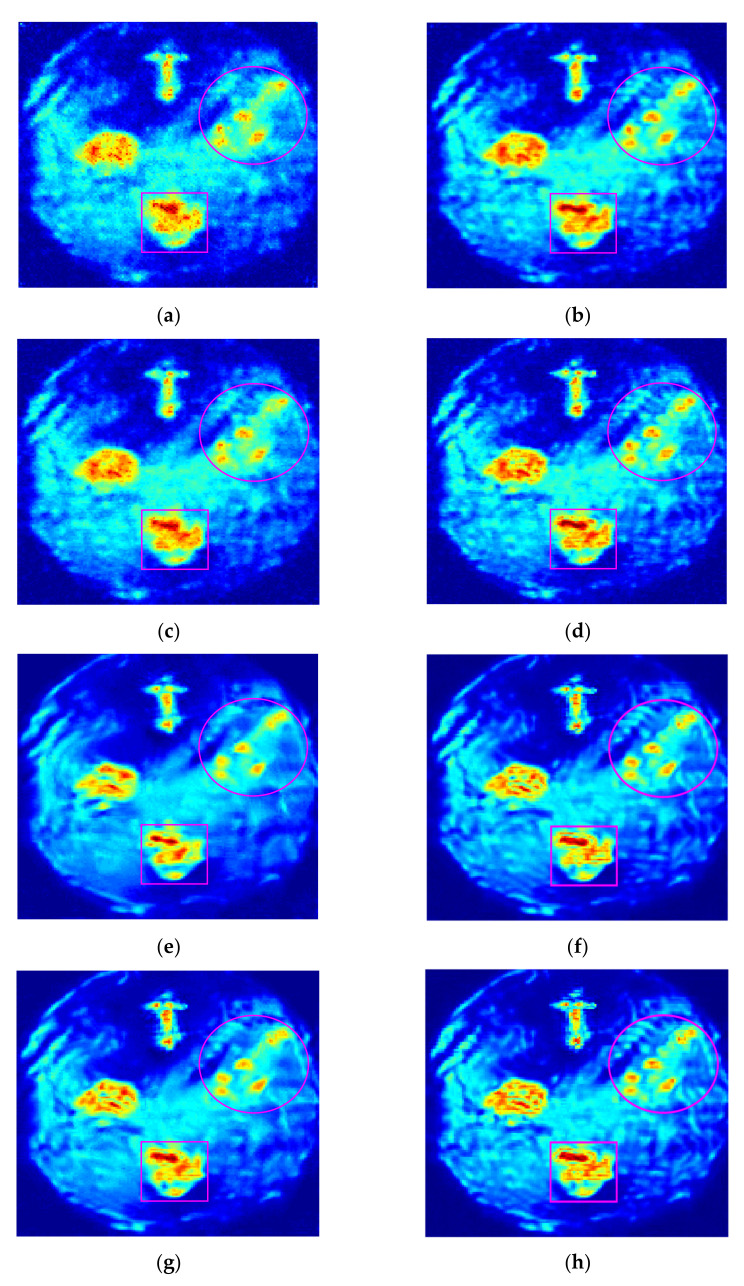
Reconstruction images of different methods for the sample at different sampling rates, and the areas marked by circles and squares are selected for detail comparison. (**a**) SSC at SR = 20%; (**b**) SSC at SR = 40%; (**c**) DSC at SR = 20%; (**d**) DSC at SR = 40%; (**e**) HSM at SR = 20%; (**f**) HSM at SR = 40%; (**g**) GSNS at SR = 20%; (**h**) GSNS at SR = 40%.

**Figure 3:**
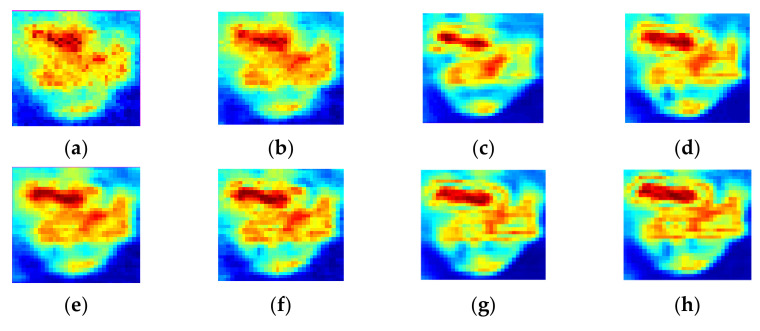
Reconstruction images of different methods for the sample at different sampling rates in the selected areas. (**a**) SSC at SR = 20%; (**b**) DSC at SR = 20%; (**c**) HSM at SR = 20%; (**d**) GSNS at SR = 20%; (**e**) SSC at SR = 40%; (**f**) DSC at SR = 40%; (**g**) HSM at SR = 40%; (**h**) GSNS at SR = 40%.

To evaluate the proposed GSNS method, we compare the GSNS with the single sparse constraint algorithm (SSC) [17], dual sparsity constraint algorithm (DSC) [18] and the hybrid sparsity model (HSM) [22]. In the experiment, all the methods were compared using the same observation matrix. The reconstructed terahertz images obtained by the four methods at different sampling rates are shown in Figure 2. Figure 2a,b show the reconstructed terahertz images of SSC when the sampling rate (SR) is 20% and 40%, respectively. Figure 2c,d are the terahertz images recovered by DSC with different sampling rates, Figure 2e,f are the terahertz images obtained by HSM, and the reconstructed terahertz images of the proposed GSNS are displayed in Figure 2g,h. When comparing Figure 2b,d,f,h, we can see that HSM and the proposed method can reconstruct the terahertz images with satisfaction at 40% SR. In particular, the terahertz image obtained by the GSNS has fewer recovery errors. However, at the same SR, the reconstructed results of DSC and SSC present some degradation and blurs. When the SR drops to 20%, it can be seen from the areas marked by circles and squares that the high under-sampling rate leads to a serious partial loss of the SSC method. For a close-up comparison, we enlarged the areas marked by squares to evaluate the image quality in Figure 3. It can be seen that the reconstruction results of DSC and HSM have some fuzzy details. Compared with the other three methods, the proposed GSNS performs well in preserving image details and edges. The terahertz image reconstructed by the proposed method is closer to the full scan terahertz image than the other two methods. Overall, GSNS obtains better results compared with the comparative methods.

**Figure 4:**
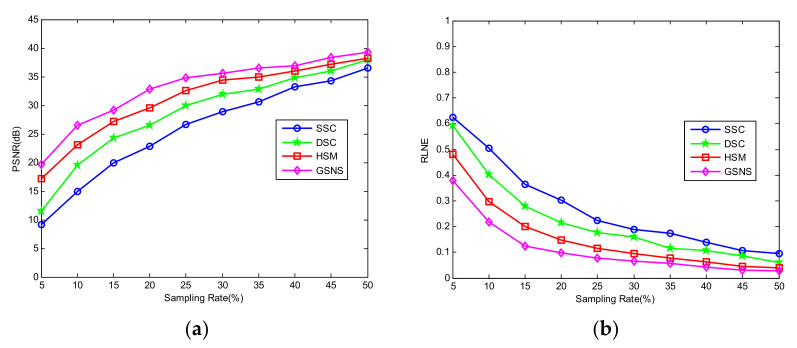
Comparison of the PSNR and RLNE curves to different sampling rates. (**a**) PSNR; (**b**) RLNE.

To further demonstrate the effectiveness of the proposed method, in Figure 4 we present the PSNR and RLNE curves of SSC, DSC, HSM and GSNS with SR from 5% to 50%. As can be seen from Figure 4, for the terahertz image with different sampling rates, the proposed GSNS has higher PSNR and lower RLNE values than the other three methods. The imaging performance of the proposed method is always better than SSC, DSC and HSM under the same SR.

The above results demonstrated that the proposed algorithm can achieve better reconstruction results. Furthermore, in order to verify the computational efficiency of the proposed algorithm, 40% SR data are used to reconstruct the terahertz image. The computation times before and after improvement are 228.1 s and 94.7 s respectively, which shows the effectiveness of the proposed algorithm.

## 4. Conclusions

In this paper, we present a fast terahertz imaging model which integrates group sparsity and nonlocal self-similarity constraints for better terahertz image reconstruction. The advantage of the proposed method is that it utilizes the structure similarity and sparsity of image patches in both two-dimensional and three-dimensional space to reconstruct the terahertz image. Moreover, an acceleration scheme based on conjugate gradient method is proposed to overcome the high computational costs of the proposed terahertz imaging model. Compared with existing methods, the experimental results show that the proposed method has better performance in detail clarity and edge preservation of the reconstructed terahertz images.

## Data Availability

Not applicable.
